# Assessment of two methods for detecting carious dentin: an in vitro study

**DOI:** 10.1186/s12903-025-05596-0

**Published:** 2025-02-19

**Authors:** Joel White, Alfa Yansane, Puja Kukreti, Pragati Nahar, Paolo Orobia, Rachel Jensen, Leslie Plack, Ram Vaderhobli, Jonathan Magnum, Larry Jenson

**Affiliations:** 1 Department of Preventive and Restorative Dental Sciences, UCSF School of Dentistry, 707 Parnassus Avenue, San Francisco, CA 94105 USA; 2 UCSF School of Dentistry, 707 Parnassus Avenue, San Francisco, CA 94105 USA; 3 Centre for Biopharmaceutical Excellence, Level 11, 655 Elizbeth St, Melbourne, 3000 Australia; 4 Incisive Technologies Pty Ltd, Level 6, 41 Exhibition Street, Melbourne, VIC 3000 Australia; 5 Richmond, Sonoma Street, CA 94805 USA

**Keywords:** Cavitated caries lesion, Caries lesion, Minimally invasive dentistry, Dentin, Detection dye

## Abstract

**Background:**

The objective of this study was to compare, in vitro, two dentinal caries lesion detector methods, Caries Finder and BlueCheck, to determine if they were substantially equivalent in their ability to aid visualization of demineralized dentin and to also to compare their performance compared to the traditional visual/tactile method of dentinal caries lesion detection in vitro.

**Methods:**

Sixty-five extracted human teeth containing lesions rated as ICDAS 4,5 or 6 were chosen and then randomly assigned to two groups. Specimens were then evaluated in standard operatory conditions by three evaluators using the traditional visual and tactile method, the Caries Finder method, and the BlueCheck method of detection. The study employed a parallel, randomized controlled study design. To test the equivalence claim, a “two-one sided test” (TOST) approach was utilized.

**Results:**

As compared to the traditional method, the Caries Finder method had a 0.9742 accuracy, 95% confidence interval [0.9578, 0.9855], 94.80% sensitivity, 98.53% specificity, 96.47% positive predictive value, 97.82% negative predictive value, 0.938 Kappa value, *p* < 2.2e-16). The BlueCheck method had a 0.9821 accuracy, 95% confidence interval [0.9682, 0.9910], 96.02% sensitivity, 99.09% specificity, 97.69% positive predictive value, 98.42% negative predictive value, 0.956 Kappa value, *p* < 2.2e-16). Inter-rater reliability and intra-rater reliability ratings were good to excellent.

**Conclusions:**

The results of this study support the conclusion that the Caries Finder and BlueCheck methods compare favorably with the traditional method of carious dentin detection. Caries Finder and BlueCheck detection methods were found to have comparable performance in their ability to differentiate carious dentin from healthy tooth structure in vitro; however further in vivo validation is required to confirm clinical equivalence. Both show good to excellent inter-rater and intra-rater reliability.

## Background

The new clinical paradigm of managing dental caries in the least invasive way has led to the quest to find new methods of detecting carious lesions earlier and more effectively than the traditional methods of detection. This in vitro study was initiated to determine substantial equivalency between a novel carious dentin lesion detection dye and a more established detection dye. It is a necessary first step towards eventual clinical trials of this new technology.

The history of using dyes to aid the visualization of carious dentin is, at best, controversial [1–5]. Dyes have been shown to lack a degree of sensitivity and specificity that would give a clinician the confidence that any dentinal structure stained by the dye was indeed “diseased” [2].

Originally intended to differentiate dentinal tissue that was infected with bacteria from tissue that was either healthy or merely demineralized, dyes were thought to be essential to the proper management of carious dentin lesions wherein all infected dentin was to be removed before restoration to prevent progression of the lesion [6, 7]. Subsequent studies showed that dyes (usually 1% acid red in propylene) did not actually detect bacteria nor did they necessarily detect denatured collagen that was beyond remineralization (another reason for tissue removal) [3, 8, 9]. Later studies supported the idea that dyes merely indicated increased porosity of the dentinal lesion [4, 10–12]. Consequently, and ironically, the use of these dyes would often lead to excess tissue removal as any area of dentin porosity would become stained. It has been shown that the potential for removing healthy or re-mineralizable tooth structure when using these dyes is significant, thus leading to complications.

In the modern paradigm of caries management, the progression of caries lesions is understood to be a function of the biofilm and oral conditions that fuel the demineralization process (caries) and not the presence of bacteria within the carious lesion [13–16]. As a result, complete removal of the carious lesion is no longer the standard of care for stopping lesion progression [17–23]. Non-restorative (non-surgical) therapies are now focused on the control of the biofilm, oral conditions, and other risk factors in order to stop lesion progression. Moreover, the use of silver diamine fluoride and other techniques has further lessened the need for extensive surgical procedures as these methods are capable of arresting and reversing demineralization without tissue removal [17]. It would seem that the use of detection dyes has little or no role to play in the new paradigm of minimally invasive caries management.

And yet, the ability to differentiate healthy from carious dentinal tissue is still important in many other ways, and detection dyes may prove to be important strategies in the new paradigm of caries management and minimally invasive dentistry. First, areas of carious dentin indicate that the caries process is ongoing. Active lesion identification (whether in enamel or dentin) is important in diagnosis and caries risk assessment in that it indicates that the patient is “out of balance” and thus at higher risk for lesion formation and progression and would benefit from therapies intended to improve that balance [24, 25]. As therapies are introduced, monitoring of those therapies is informed by the presence or absence of active lesions. Detection dyes have the potential to contribute to the monitoring of lesion activity, the effectiveness of therapeutic interventions and patient education efforts, though future in vivo research is needed to validate these roles. Second, the traditional method for identifying active dentin lesions utilizes visualization and probing with a dental explorer. This method is limited in several ways. Visualization is limited by the large variance in color and appearance of demineralized and healthy dentin [26]. The use of a dental explorer to determine demineralization is limited in two ways; studies have shown that the sensitivity of this method is poor [27] and that using it risks exacerbating the carious process [28–32]. It is generally accepted that aggressive probing with a sharp instrument for enamel or dentinal lesions is contraindicated as a routine procedure. Lastly, the presence of carious dentinal tissue can indicate an area that might thwart restorative goals [19]. It is well known that demineralized dentin can undermine enamel structure and existing restorations. It has also been shown that demineralized dentin can compromise the marginal seal and retention of restorations dependent on adhesive techniques, and if there is extensive demineralization, the compressive strength of a restoration can be diminished [33–36]. Safer and more effective methods of detecting demineralized dentin are clearly needed in the new paradigm of caries management.

The study presented here was an opportunity to investigate and compare the carious dentin-detecting ability of two detection dyes: a traditional dye, Caries Finder (CF) and a novel dye, BlueCheck (BC). Each was compared to the traditional method (TM) of carious dentin detection using unaided visualization and tactilization. The Caries Finder method and the BlueCheck method were also compared to each other to determine if they were substantially equivalent in their ability to aid visualization of carious dentin in vitro. The traditional method of evaluating dentin lesions was used in the study as a gold standard. Despite several technologies that have been introduced to improve lesion detection, the traditional method is still the most commonly used method for dentin lesion evaluation [1, 26, 37].

Caries Finder (Danville Materials, San Ramon, CA) is a patented, contemporary version of the time-tested solution of either 1% acid red or 1% FD&C green in propylene glycol. Caries Finder is intended to be used in cavity preparation to identify carious dentin. The manufacturer states that this product stains the collagen that is exposed in the carious process of demineralization. It is postulated that the dye molecules have a unique affinity for loose collagen present within areas of demineralized dentin.

BlueCheck (Incisive Technologies Pty Ltd, Melbourne, Victoria, Australia) is a new product for detecting demineralized dentin and is intended to aid the visualization of carious lesions in enamel and dentin. It is a solution that is intended as a “porosity probe” applied directly to a tooth to identify areas of demineralization in both enamel and dentin and hypo-mineralized dental tissues under white light in standard clinical environments. BlueCheck solution contains an engineered biomolecule that consists of a deep-blue dye (Amido black) linked to a protein (hemoglobin) that has a specific affinity for porous hydroxyapatite. BlueCheck utilizes the natural hydroxyapatite-binding chemistry of proteins to specifically and reversibly bind to porous dental hydroxyapatite. It does not rely upon the presence of bacteria, acid/bacterial byproducts, or collagen. The intensity of the staining is correlated to the degree of demineralization [38]. BlueCheck is intended to be applied to teeth at an initial examination to reveal areas of demineralization in enamel and dentin and to be used to monitor lesions following therapeutic efforts. It also has the potential to be used during cavity preparation to reveal areas that are demineralized. BlueCheck is fully reversible and can be easily removed by following the instructions for use.

Both BlueCheck and Caries Finder stain demineralized dentin progressively with darker color indicating a higher degree of demineralization. Both dyes are applied following cleansing the tooth of plaque/biofilm. The application process for both dyes requires minimal training and both can be used in any clinical environment where there is adequate lighting and water supply.

The objective of this study was to compare the carious dentin lesion detection ability of the BlueCheck method and the Caries Finder method to the traditional method of carious dentin lesion detection, and to each other through the sensitivity (Se), specificity (Sp), positive predictive value (PPV), and negative predictive value (NPV) of each method. Inter-rater and intra-rater reliabilities for each method were also assessed.

## Methods

### Specimen selection, randomization, and assignment to groups

Sixty-five specimens of extracted human permanent teeth with cavitated smooth surface carious lesions were selected by three dentists trained in the International Caries Detection and Assessment System (ICDAS) from a biobank of teeth at the University of California, San Francisco School of Dentistry. Specimens were previously sterilized in an autoclave and stored in 0.1% thymol aqueous solution. Agreement of ICDAS score by 2 of 3 dentists was required for specimen inclusion in the study. All selected teeth had one smooth surface lesion that met the ICDAS 4, 5, or 6 criteria for cavitated lesions: visually evident enamel breakdown with indications of dentin involvement [39]. Specimens were required to have lesions that had a periphery of either healthy enamel (ICDAS 0) or early enamel disease (ICDAS 1 or 2). Specimens were randomized by computer assignment and then assigned into 2 groups (BC and CF) with near-equal numbers of specimens. Each specimen was assigned a unique identification code.

### Preparation, photography, and grid placement

**Fig. 1:**
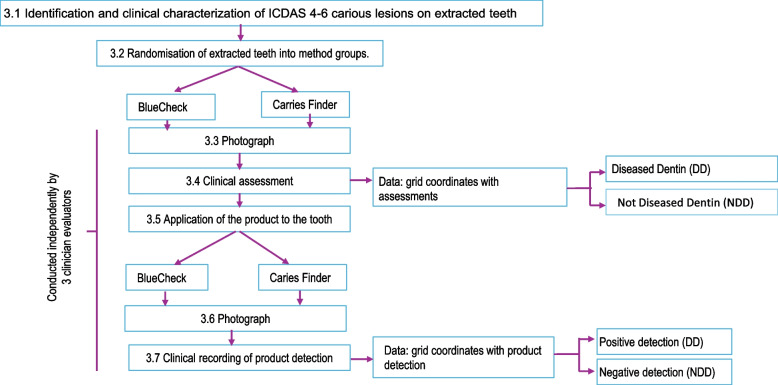
Flow chart showing the workflow for the comparison of BC and CF versus TM

For evaluation, each specimen was cleaned and dried and then placed in a rigid wax mold and photographed at a repeatable distance. The resulting images were digitally overlaid with a standardized (2 mm × 2 mm) positional grid pattern demarcating areas to be examined (squares). Each specimen and its corresponding photograph were then evaluated by all three examiners and scored. These scores became the gold standard for the investigation. Scoring was determined by the presence or absence of “diseased” (demineralized/carious) dentin within any given grid area (square): DD for diseased dentin and NDD for not diseased dentin. A square that had diseased cementum or enamel or healthy enamel was designated as NDD. Any square that could not be determined to be either DD or NDD was not included in the analysis and a red “x” was placed in that square for the next round of evaluations using BC or CF. BC or CF was then applied to the teeth as per group assignment and as per the manufacturer’s instructions. Specimens were then returned to their rigid wax molds and photographed again at the original distance. The resultant images were then digitally overlaid with the original grid pattern that was used to evaluate TM, and then evaluated and scored. Figure 1 shows the experimental sequence and data collection workflow.

### Examiners, calibration and scoring

Three experienced dental clinicians, trained and calibrated in ICDAS, were asked to be examiners for the study which was conducted in a standard clinical setting with available operatory lights and a computer screen for displaying the comparison images. Examiners were calibrated for the study using specimens outside of those selected for the study. Calibration of examiners included a review of literature and the examination of photographs of healthy and diseased enamel and dentin that have been stained. Calibration of presence or absence of stain determination occurred during pilot studies with review of concordant and discordant observations by examiners.

Examiners were provided with specimens, a dental mirror, a dental explorer, periodontal probes, loupes, and compressed air syringes. They were allowed to handle and manipulate the specimens as they viewed the comparison images on the computer screen. For the first round of evaluation, examiners independently scored the image grid for each specimen without BC or CF applied. These scores became the standard against which scores for BC and CF were compared. For the second round of evaluations, one designated evaluator applied BC to the specimens in that group and then all examiners would independently score the image grid squares for those specimens. For the third round of evaluations, the same designated examiner would apply CF to the specimens in that group and then all evaluators would independently score the image grid squares for that group of specimens.

**Fig. 2:**
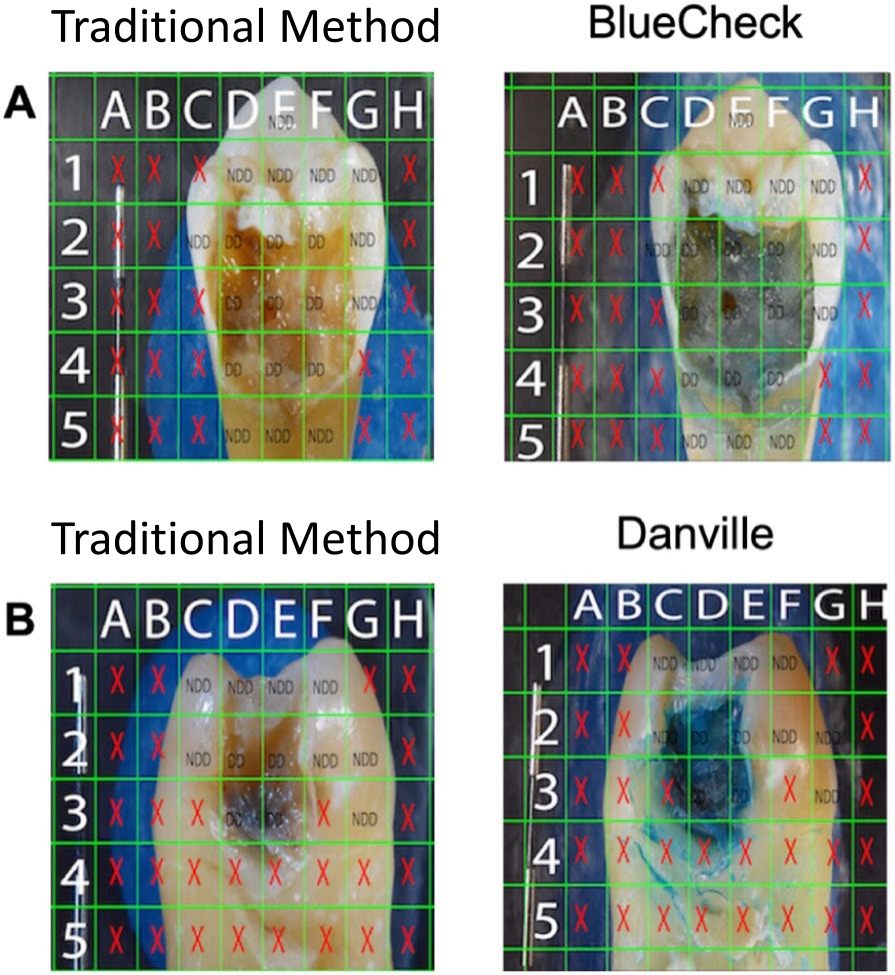
A is a photograph of an untreated specimen before and after the application of BC with an overlaid grid and scores of either DD or NDD. B is a photograph of another specimen before and after the application of CF with an overlaid grid and scores of either DD or NDD. Grid areas with a red X were not scored

Examiners scored each square of the grid for each specimen for diseased dentin (DD) or not diseased dentin (NDD). A positive determination of disease required a predominance (≥ 50%) of disease within the square. Figure 2 shows examples of typical grided images for TM, BC and CF. Squares containing a red X were not scored. For the test–retest assessment, each examiner repeated their scoring on four randomly selected specimens within each group.

### Statistical methods/analysis

#### Sample size calculation

We employed a parallel, randomized controlled study design with a 1 to 1, BC to CF ratio to evaluate the equivalence of the methods. The unit of randomization was the dental tooth. All eligible teeth were simultaneously randomized by computer-generated assignment at the time of the trial. The analysis estimated the proportion of detection using the two methods BC and CF. The sample size/power calculations were based on the estimated number of teeth cleaned, imaged and available for analysis. To perform the power analysis, investigators reported the detectable difference for the fixed sample size of 65 teeth and an assumed 90% power. The total sample of 65 teeth yielded 1,215 (CF: 590, BC: 625) square sized surfaces for demineralization detection by reviewers. There were 8 unusable surfaces found among the CF cohort and 10 among the BC cohort yielding a final sample of 65 teeth and 1197 surfaces (CF: 582, BC: 615). Given the 65 teeth and the 1197 surfaces, an assumed power to detect differences of 90%, a standard significance level of 0.05, the two one-sided test will be able to detect a difference of 0.09.

#### Examiner reliability

To determine intra-examiner reliability, 10% of samples were re-evaluated a second time by each examiner.

### Statistical methods

The diseased dentin outcome was measured as a binary variable: presence or absence of staining with clinician evaluator assessment as stained indicating carious lesion. To test the equivalence claim in the trial comparing two different methods for carious lesion detection, a “two-one-sided test” (TOST) approach was utilized [40]. We report the proportion of diseased dentin with both the BC and CF methods along with their absolute difference. The equivalence margin is set at 15% (delta = 15.0%). A two-sided 90% confidence interval was used to establish whether equivalence is satisfied at the 5% significance level. Additionally, the sensitivity of both BC and CF in the detection of diseased dentin was compared using the same two-one-sided test approach. If the result confidence limits include the given 15% delta, then there is evidence that the two methods are equivalent.

The traditional method, with no intervention compared to the two interventions, was used to calculate sensitivity. For TM, no intervention on all teeth before the application of intervention establishes the gold standard with a 2/3 evaluator agreement for each grid assessment. These statistical methods to determine sensitivity were used for the healthy dentin collected. The same methodology was utilized to determine sensitivity, positive predictive value (PPV) and negative predictive value (NPV).

## Results

### Descriptive statistics

A total of 65 teeth containing 70 lesions were examined and scored for each square. The total number of squares examined for all methods was 1197 with 582 squares for CF and 615 squares for BC. Tables 1 and 2 show an overview of the data used for statistical analysis.

**Table 1:** Total number of squares evaluated by method and group

Total Teeth	65	
Total number of lesions	70	
Total number of squares evaluated that met the inclusion criteria	1197	
Total number of squares evaluated by method	TM	1197
	CF	582
	BC	615

**Table 2:** Total squares evaluated as DD and NDD by method and group

Number of squares evaluated as diseased dentin (DD) by method and group	CF group		BC group	
	CF	TM	BC	TM
	164	173	169	176
Number of squares evaluated as not-diseased dentin (NDD) by method and group	403	409	435	439

### Statistical analysis: Two-One-Sided Approach (TOST)

**Fig. 3:**
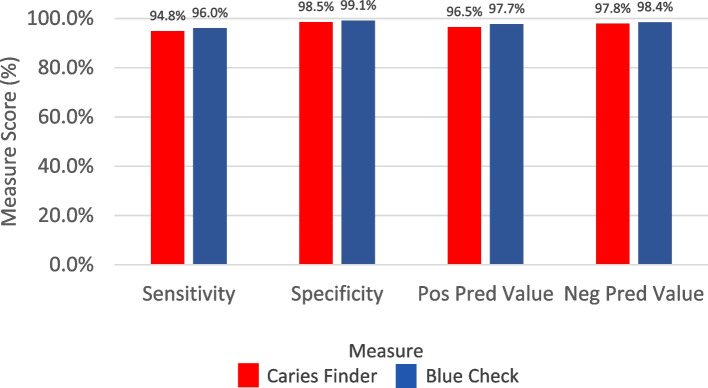
Measure scores of sensitivity, specificity, positive predictive value, and negative predictive value of BC and CF compared to the gold standard, TM

**Fig. 4:**
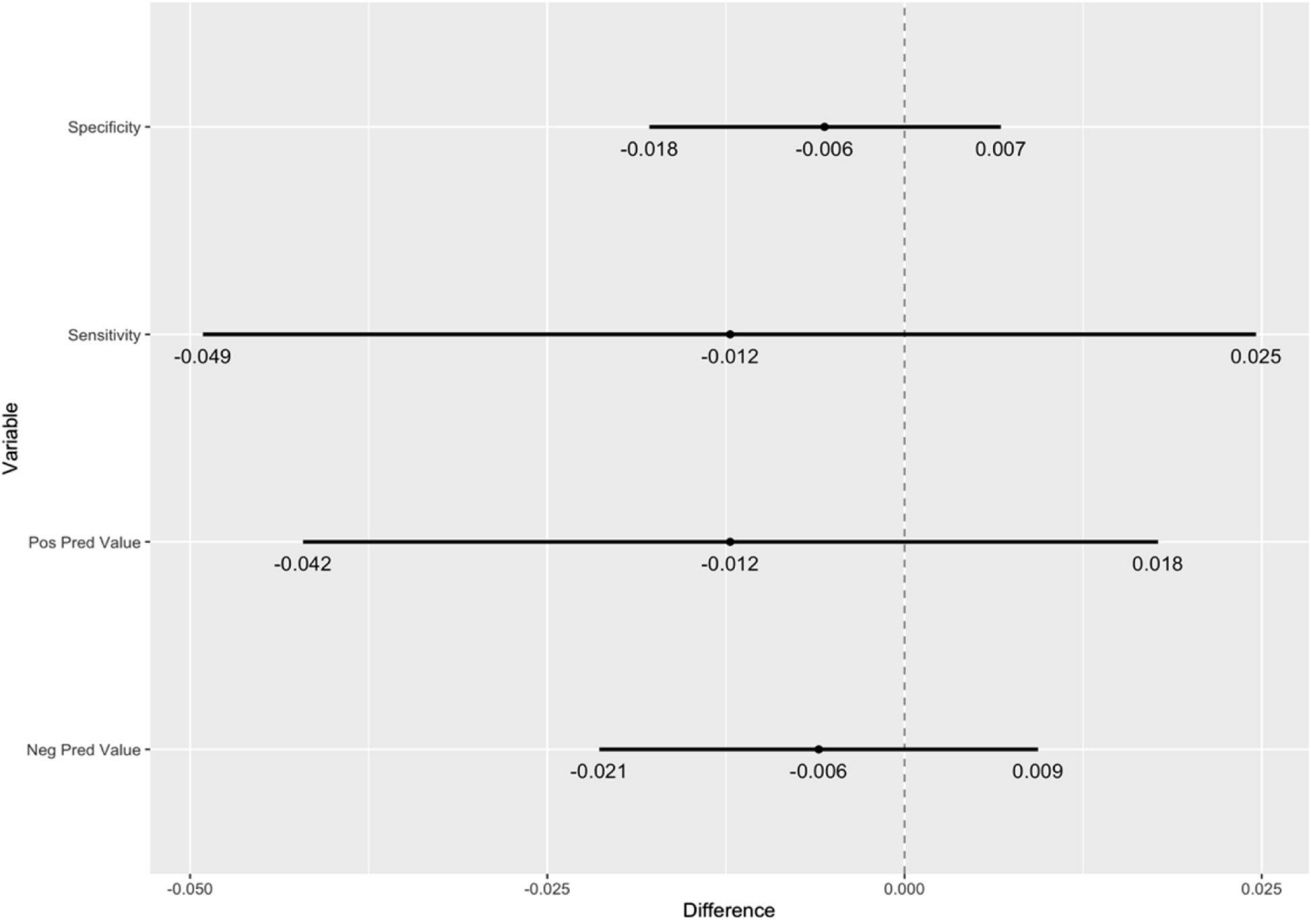
95% confidence interval of specificity, sensitivity, positive predictive value and negative predictive value outcome difference between BC and CF

There were 164 out of 173 measurement areas accurately (as compared to TM) diagnosed as diseased dentin using CF: 0.9742 accuracy, 95% confidence interval [0.9578, 0.9855], 94.80% sensitivity, 98.53% specificity, 96.47% positive predictive value, 97.82% negative predictive value, 0.938 Kappa value, *p* < 2.2e-16). Using BC, 169 out of 176 diseased dentin measurements were accurately diagnosed using BC: 0.9821 accuracy, 95% confidence interval [0.9682, 0.9910], 96.02% sensitivity, 99.09% specificity, 97.69% positive predictive value, 98.42% negative predictive value, 0.956 Kappa value, *p* < 2.2e-16). Figure 3 displays the measure scores of BC and CF compared to the gold standard TM. It shows that the BC and CF methods are comparable to the reference standard (TM) in their ability to differentiate diseased dentin from non-diseased dentin. Sensitivity, specificity, NPV and PPV values for BC and CF are all above 94%. Figure 4 shows that BC is substantially equivalent (non-inferior) to CF.

### Reliability

Tables 3 and 4 are summaries of inter-rater and intra-rater reliability analyses.

**Table 3:** Summary of the inter-rater reliability analysis

Inter-Rater Reliability by Method			
**TM**			
	Value	Lower Bound	Upper Bound
ICC (Absolute Agreement)	0.830	0.812	0.847
ICC (Consistency)	0.835	0.820	0.849
**CF**			
	Value	Lower Bound	Upper Bound
ICC (Absolute Agreement)	0.821	0.796	0.844
ICC (Consistency)	0.826	0.803	0.846
**BC**			
	Value	Lower Bound	Upper Bound
ICC (Absolute Agreement)	0.876	0.860	0.891
ICC (Consistency)	0.876	0.860	0.891

**Table 4:** Summary of intra-rater reliability analysis with lower and upper bounds of confidence interval

Intra-Rater Reliability			
	**Examiner 1**	**Examiner 2**	**Examiner 3**
**TM**			
ICC (Absolute Agreement)	0.889 (0.823, 0.931)	0.939 (0.913, 0.957)	0.887 (0.820, 0.930)
ICC (Consistency)	0.888 (0.822, 0.931)	0.938 (0.912, 0.957)	0.889 (0.822, 0.931)
**CF**			
ICC (Absolute Agreement)	0.954 (0.923, 0.972)	0.788 (0.651, 0.873)	0.965 (0.928, 0.983)
ICC (Consistency)	0.954 (0.925, 0.973)	0.800 (0.677, 0.880)	0.965 (0.928, 0.983)
**BC**			
ICC (Absolute Agreement)	0.890 (0.815, 0.934)	1.000	0.877 (0.775, 0.935)
ICC (Consistency)	0.898 (0.835, 0.937)	1.000	0.883 (0.787, 0.938)

These results show that both the BlueCheck method of detection and the Caries Finder method of detection compares favorably to the traditional method of carious dentin detection. As for the inter-rater reliability of the methods studied, Table 3 shows good agreement between evaluators for BC, CF, and TM. Values above 0.75 are good according to the guidelines from Landis and Koch 1977 [41]. Table 4 shows good to excellent intra-rater reliability.

## Discussion

The ability to clinically determine the existence and extent of carious dentin is essential to modern caries management strategies. Even though the traditional method of carious dentin detection is still the most commonly used, it suffers from less-than-ideal reliability and involves the use of a dental explore that can further damage carious tissue. Safer and more effective methods of carious dentin detection are being sought. This study was an opportunity to investigate a novel method of detection and compare it to two of the methods currently being used. The results of this study support the conclusion that the BlueCheck method of detection compares favorably to the traditional and the Caries Finder methods of detection.

When interpreting the findings of studies like this one, sensitivity and specificity values must be considered in the context of what the dye seeks to disclose. The assumption in this study is that these two methods seek to identify dentin that has been demineralized by the carious process. It is entirely possible that all three methods also identify areas of dentin that are hypo-mineralized by some other process. This study is a comparison study only: the results show that both the BlueCheck and Caries Finder methods are at least as good as the traditional method and at least as good as each other. This is a necessary first step towards future studies that might establish the superiority of the methods used here. We are clear that none of the methods studied seek to detect caries. It is unfortunate that the word “caries” has been used to refer to both the disease process and the demineralization of tissues that the caries process creates. This conflation of meanings has led to meaningless terms such as “residual caries”, ambiguous terms such as “recurrent caries”, and dyes being erroneously referred to as “caries detectors”. Caries is a diagnosis made by a dentist considering all clinical findings: no dye can do this directly. At best, dyes can give information to the dentist that informs that diagnosis. We suggest that a caries diagnosis indicates an ongoing process of demineralization. Tooth tissues that evidence demineralization can, though not always, indicate that the process is active. We know that in the absence of the demineralization process, enamel and dentin will remineralize and form an impermeable layer. We also know that detection dyes like Caries Finder and BlueCheck will generally fail to stain structures that have an impermeable layer of remineralization [38, 42]. In the new paradigm of caries management, detection dyes can be valuable if their intended target is an area of demineralization suspected to be caused by the caries process and the dentist only utilizes this information within the context of clear therapeutic aims: diagnostic, preventative, surgical and/or restorative.

This study is limited in that it is an in vitro study and results may be different when either of the two methods is used clinically. Future in vivo studies are needed to fully evaluate the clinical effectiveness of either method**.** In vivo studies are a necessary direction for future study. The use of the TM as a gold standard is limited and future studies that employ gold standards such as histological analysis and those that quantify the extent of demineralization would give a clearer assessment of the efficacy of the two methods examined here. No attempt was made to differentiate between demineralized dentin caused by a disease process and naturally occurring demineralized areas such as those found near the DEJ and peri pulpally. No attempt was made to quantitatively correlate the intensity of the staining with the extent of demineralization; this would also be a good direction for future studies. Given studies like Kidd 1989 [43] showing that, as compared to detection dyes, the traditional method fails to detect all demineralized tissue, we might expect a high level of false positives leading to lower values of specificity. This was not the case in this study: specificity values were high for both dyes.

The clinical significance of the results presented here is that both methods of dye detection are equivalent and compare favorably to the traditional method. Thus, dentists now have a variety of methods available for carious dentin detection. It may be that each method has clinical advantages over the others. For example, using either BlueCheck or Caries Finder instead of the traditional method may significantly lower the need for probing dentin with a sharp explorer. As mentioned above, knowing the mineralization state of the dentin may prove to be an essential aid to caries risk assessment, caries diagnosis, monitoring therapeutic interventions and restorative success. Moreover, the ability to visualize demineralization more clearly could be beneficial for patient education efforts and the training of new dentists.

There are many aspects of the two products studied here that could be pursued in future studies. An in vivo study could focus on the effects of oral conditions (saliva, plaque, etc.) and patient comfort and sensitivity. A usability study could address issues of provider training, ease of application, cost-effectiveness, best practices, patient education and effective disposal of surplus product. Long-term studies could investigate the effects these dyes have on restoration and tooth survival as well as success at monitoring lesions over time. Another study could examine the benefit of combining the detection dyes with other modalities such as DIAGNOdent, optical coherence tomography or bioluminescence and relate positive detection to staining intensity. Still another study could pursue the integration, benefits and economics of these products in large-scale public health initiatives and use in pediatric dentistry.

There is nothing automatic about using carious dentin dyes; it is still up to the dentist to decide if, where and how much dentin might need to be removed to achieve treatment goals [23]. And, it is still up to the dentist to make a diagnosis of “caries”: an assessment that the patient is experiencing an ongoing process of tooth demineralization due to acids produced by bacteria and not by some other pathological process. For example, both methods would presumably identify areas of dentin that were demineralized due to acid erosion. Detection dyes merely help indicate the presence of porosity: permeable, demineralized enamel and dentin. Healthy dentin can include areas of reduced mineralization (pulpal and DEJ areas) and so the use of these dyes requires expert clinical judgment to determine the proper management of the lesion and maximize their potential in the new paradigm of minimally invasive caries management. The two dyes assessed in this study can best be utilized to *confirm* the initial clinical judgment of the dentist.

## Conclusions

The results of this study support the conclusion that Caries Finder and BlueCheck, intended as aids to the visualization of demineralized dentin, compare favorably with the traditional method of demineralized dentin detection. The results suggest that BC and CF have comparable performance in vitro to differentiate carious dentin from healthy tooth structure.; however, further in vivo validation is required to confirm clinical equivalence. Both CF and BC show good to excellent inter-rater and intra-rater reliability. These findings indicate that BC and CF are promising methods for detecting demineralized dentin in vitro. Further studies, particularly in vivo investigations, are necessary to validate their clinical applicability within the paradigm of minimally invasive dentistry. Both the Caries Finder method and the BlueCheck methods of detection may play an important role in the new paradigm of minimally invasive dentistry when diagnostic and therapeutic goals are clearly articulated. These methods have the potential to contribute to minimally invasive dentistry, particularly for aiding visualization of demineralized dentin. However, clinical trials are needed to confirm their practical utility and impact on patient outcomes. Future in vivo studies will build an understanding of their use in clinical practice within the minimally invasive paradigm of caries management**.**

## Data Availability

The datasets used and/or analysed during the current study are available from the corresponding author on reasonable request.

## Abbreviations

CF: Caries Finder
BC: BlueCheck
TM: Traditional Method
Se: Sensitivity
Sp: Specificity
PPV: Positive Predictive Value
NPV: Negative Predictive Value
ICDAS: International Caries Detection and Assessment System
DD: Diseased Dentin
NDD: Not Diseased Dentin
TOST: Two-One-Sided-Test
