# Correction: Assessment of two methods for detecting carious dentin: an in vitro study

**DOI:** 10.1186/s12903-025-05740-w

**Published:** 2025-03-13

**Authors:** Joel White, Alfa Yansane, Puja Kukreti, Pragati Nahar, Paolo Orobia, Rachel Jensen, Leslie Plack, Ram Vaderhobli, Jonathan Mangum, Larry Jenson

**Affiliations:** 1 Department of Preventive and Restorative Dental Sciences, UCSF School of Dentistry, 707 Parnassus Avenue, San Francisco, CA 94105 USA; 2 UCSF School of Dentistry, 707 Parnassus Avenue, San Francisco, CA 94105 USA; 3 Centre for Biopharmaceutical Excellence, Level 11, 655 Elizbeth St, Melbourne, 3000 Australia; 4 Incisive Technologies Pty Ltd, Level 6, 41 Exhibition Street, Melbourne, VIC 3000 Australia; 5 Richmond, Sonoma Street, CA 94805 USA

**Correction: BMC Oral Health (2025) 25:258**

10.1186/s12903-025-05596-0

In this article [1], the author’s name Jonathan Mangum was incorrectly written as Jonathan Magnum.
